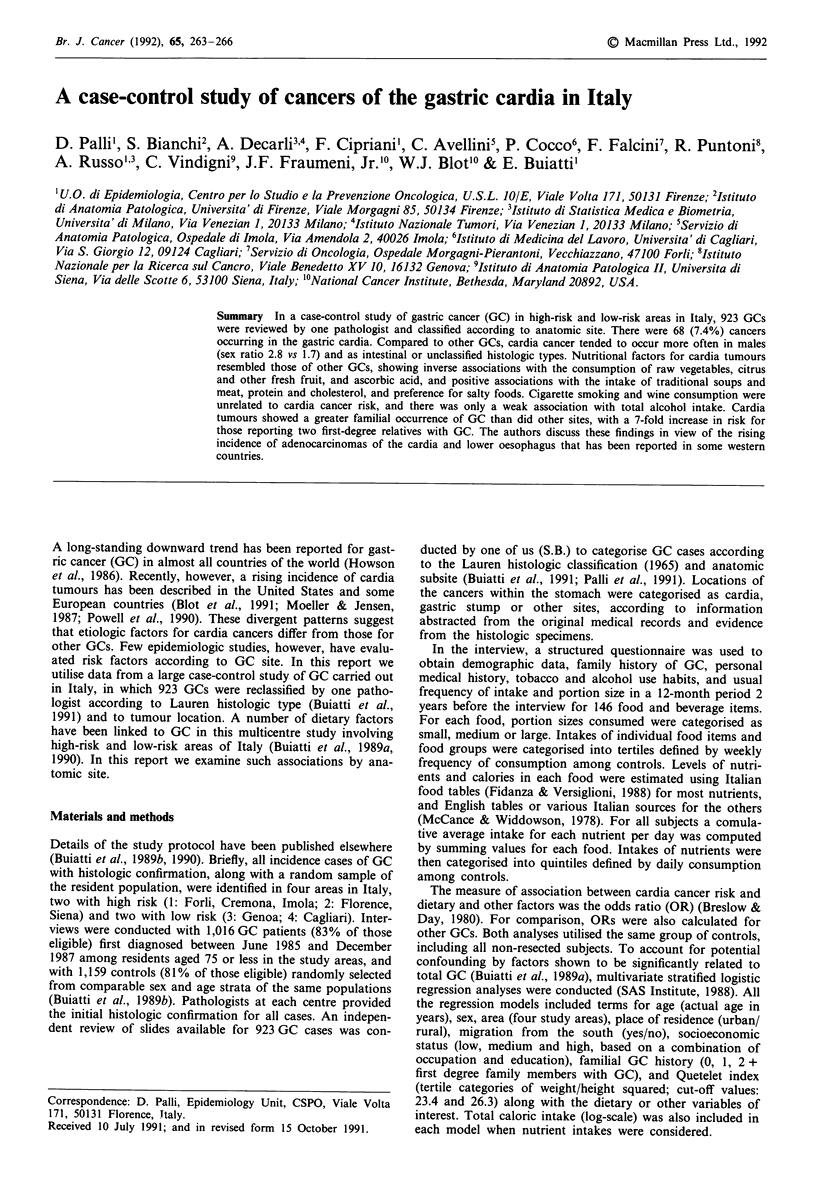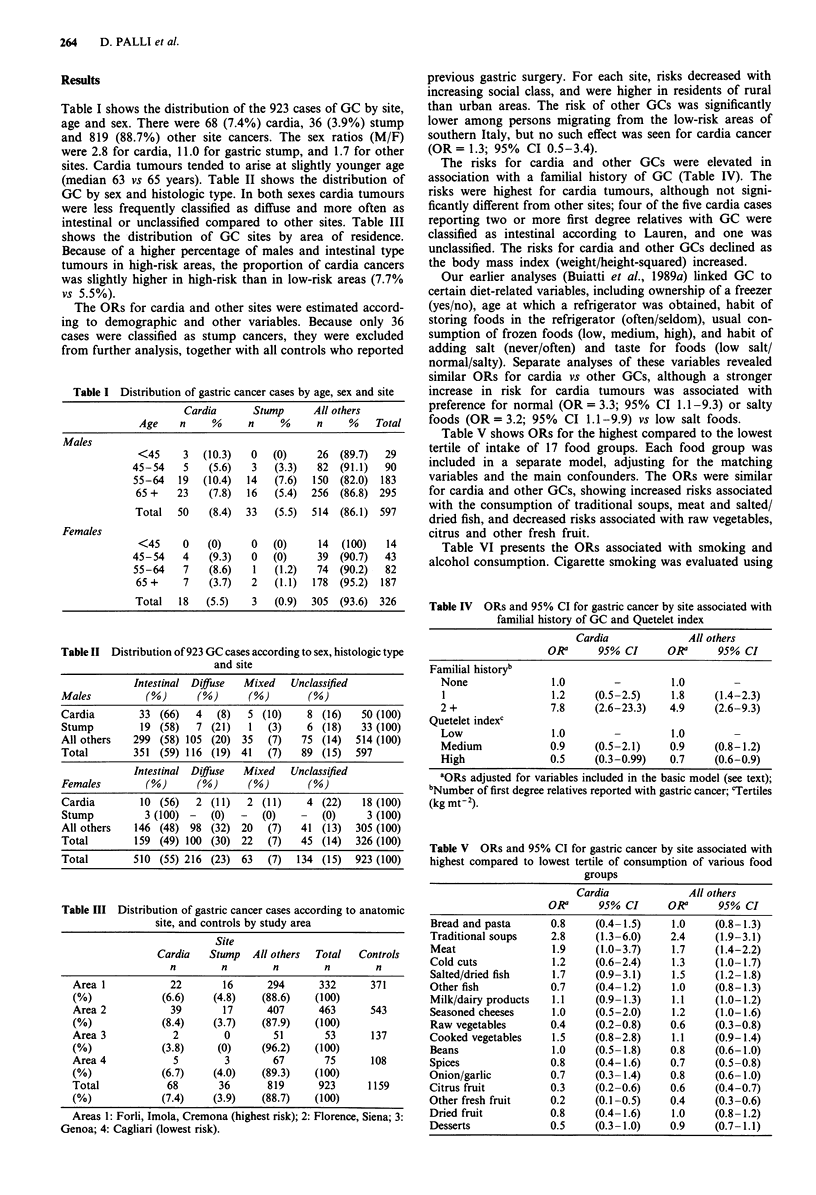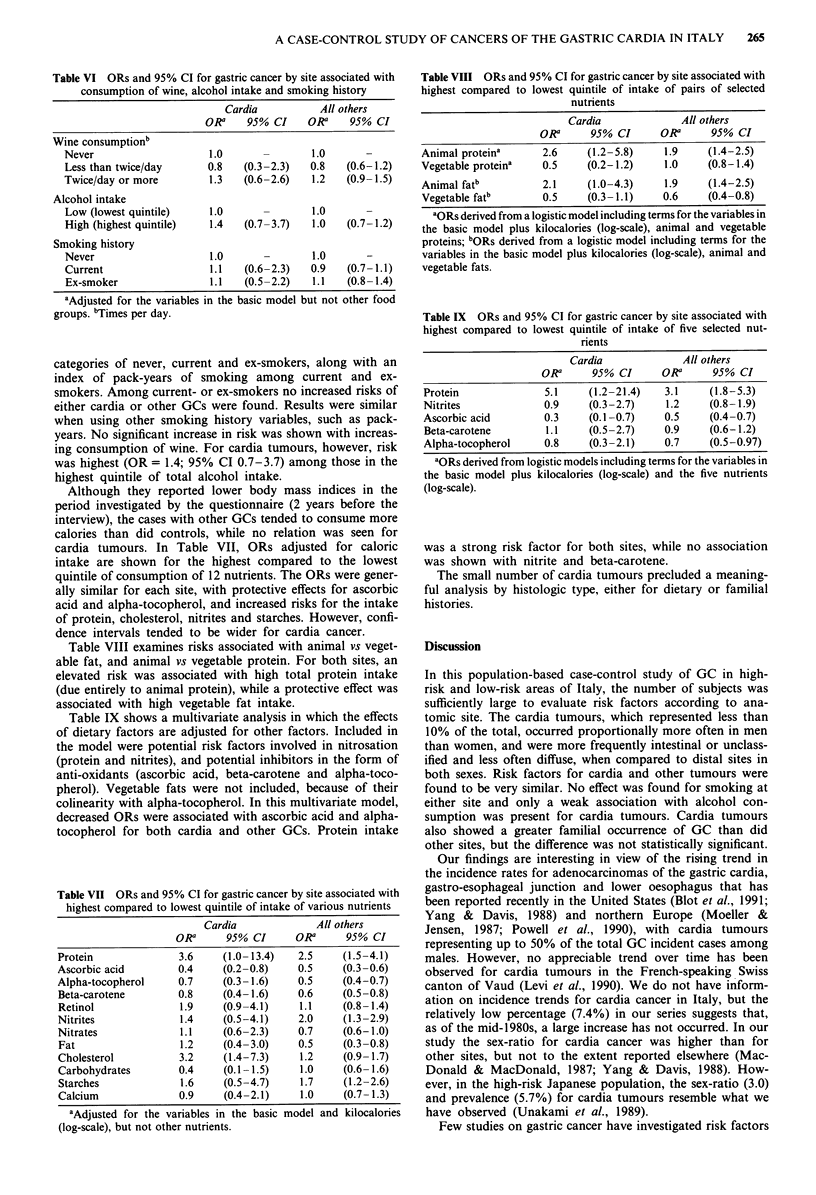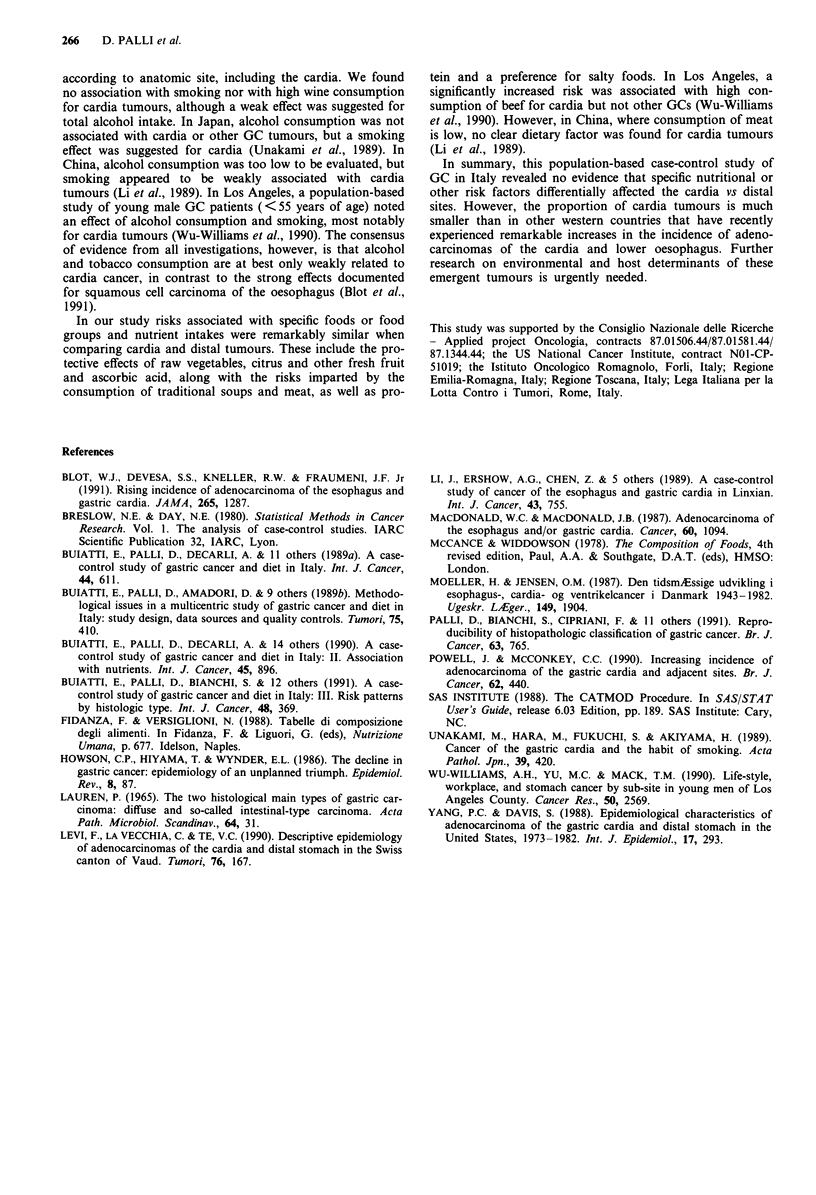# A case-control study of cancers of the gastric cardia in Italy.

**DOI:** 10.1038/bjc.1992.52

**Published:** 1992-02

**Authors:** D. Palli, S. Bianchi, A. Decarli, F. Cipriani, C. Avellini, P. Cocco, F. Falcini, R. Puntoni, A. Russo, C. Vindigni

**Affiliations:** U.O. di Epidemiologia, Centro per lo Studio e la Prevenzione Oncologica, Firenze, Italy.

## Abstract

In a case-control study of gastric cancer (GC) in high-risk and low-risk areas in Italy, 923 GCs were reviewed by one pathologist and classified according to anatomic site. There were 68 (7.4%) cancers occurring in the gastric cardia. Compared to other GCs, cardia cancer tended to occur more often in males (sex ratio 2.8 vs 1.7) and as intestinal or unclassified histologic types. Nutritional factors for cardia tumours resembled those of other GCs, showing inverse associations with the consumption of raw vegetables, citrus and other fresh fruit, and ascorbic acid, and positive associations with the intake of traditional soups and meat, protein and cholesterol, and preference for salty foods. Cigarette smoking and wine consumption were unrelated to cardia cancer risk, and there was only a weak association with total alcohol intake. Cardia tumours showed a greater familial occurrence of GC than did other sites, with a 7-fold increase in risk for those reporting two first-degree relatives with GC. The authors discuss these findings in view of the rising incidence of adenocarcinomas of the cardia and lower oesophagus that has been reported in some western countries.


Br. J. Cancer (1992), 65, 263-266 © Macmillan Press Ltd., 1992

### A case-control study of cancers of the gastric cardia in Italy

D. Palli[1], S. Bianchi[2], A. Decarli[3,4], F. Cipriani[1], C. Avellini[5], P. Cocco[6], F. Falcini[7], R. Puntoni[8], A. Russo[1,3], C. Vindigni[9], J.F. Fraumeni, Jr.[10], W.J. Blot[10] & E. Buiatti[1]

[1]*U.O. di Epidemiologia, Centro per lo Studio e la Prevenzione Oncologica, U.S.L. 10/E, Viale Volta 171, 50131 Firenze;* [2]*Istituto di Anatomia Patologica, Universita' di Firenze, Viale Morgagni 85, 50134 Firenze;* [3]*Istituto di Statistica Medica e Biometria, Universita' di Milano, Via Venezian 1, 20133 Milano;* [4]*Istituto Nazionale Tumori, Via Venezian 1, 20133 Milano;* [5]*Servizio di Anatomia Patologica, Ospedale di Imola, Via Amendola 2, 40026 Imola;* [6]*Istituto di Medicina del Lavoro, Universita' di Cagliari, Via S. Giorgio 12, 09124 Cagliari;* [7]*Servizio di Oncologia, Ospedale Morgagni-Pierantoni, Vecchiazzano, 47100 Forli;* [8]*Istituto Nazionale per la Ricerca sul Cancro, Viale Benedetto XV 10, 16132 Genova;* [9]*Istituto di Anatomia Patologica II, Universita di Siena, Via delle Scotte 6, 53100 Siena, Italy;* [10]*National Cancer Institute, Bethesda, Maryland 20892, USA.*

**Summary** In a case-control study of gastric cancer (GC) in high-risk and low-risk areas in Italy, 923 GCs were reviewed by one pathologist and classified according to anatomic site. There were 68 (7.4%) cancers occurring in the gastric cardia. Compared to other GCs, cardia cancer tended to occur more often in males (sex ratio 2.8 *vs* 1.7) and as intestinal or unclassified histologic types. Nutritional factors for cardia tumours resembled those of other GCs, showing inverse associations with the consumption of raw vegetables, citrus and other fresh fruit, and ascorbic acid, and positive associations with the intake of traditional soups and meat, protein and cholesterol, and preference for salty foods. Cigarette smoking and wine consumption were unrelated to cardia cancer risk, and there was only a weak association with total alcohol intake. Cardia tumours showed a greater familial occurrence of GC than did other sites, with a 7-fold increase in risk for those reporting two first-degree relatives with GC. The authors discuss these findings in view of the rising incidence of adenocarcinomas of the cardia and lower oesophagus that has been reported in some western countries.

A long-standing downward trend has been reported for gastric cancer (GC) in almost all countries of the world (Howson *et al.*, 1986). Recently, however, a rising incidence of cardia tumours has been described in the United States and some European countries (Blot *et al.*, 1991; Moeller & Jensen, 1987; Powell *et al.*, 1990). These divergent patterns suggest that etiologic factors for cardia cancers differ from those for other GCs. Few epidemiologic studies, however, have evaluated risk factors according to GC site. In this report we utilise data from a large case-control study of GC carried out in Italy, in which 923 GCs were reclassified by one pathologist according to Lauren histologic type (Buiatti *et al.*, 1991) and to tumour location. A number of dietary factors have been linked to GC in this multicentre study involving high-risk and low-risk areas of Italy (Buiatti *et al.*, 1989*a*, 1990). In this report we examine such associations by anatomic site.

### Materials and methods

Details of the study protocol have been published elsewhere (Buiatti *et al.*, 1989*b*, 1990). Briefly, all incidence cases of GC with histologic confirmation, along with a random sample of the resident population, were identified in four areas in Italy, two with high risk (1: Forli, Cremona, Imola; 2: Florence, Siena) and two with low risk (3: Genoa; 4: Cagliari). Interviews were conducted with 1,016 GC patients (83% of those eligible) first diagnosed between June 1985 and December 1987 among residents aged 75 or less in the study areas, and with 1,159 controls (81% of those eligible) randomly selected from comparable sex and age strata of the same populations (Buiatti *et al.*, 1989*b*). Pathologists at each centre provided the initial histologic confirmation for all cases. An independent review of slides available for 923 GC cases was conducted by one of us (S.B.) to categorise GC cases according to the Lauren histologic classification (1965) and anatomic subsite (Buiatti *et al.*, 1991; Palli *et al.*, 1991). Locations of the cancers within the stomach were categorised as cardia, gastric stump or other sites, according to information abstracted from the original medical records and evidence from the histologic specimens.

In the interview, a structured questionnaire was used to obtain demographic data, family history of GC, personal medical history, tobacco and alcohol use habits, and usual frequency of intake and portion size in a 12-month period 2 years before the interview for 146 food and beverage items. For each food, portion sizes consumed were categorised as small, medium or large. Intakes of individual food items and food groups were categorised into tertiles defined by weekly frequency of consumption among controls. Levels of nutrients and calories in each food were estimated using Italian food tables (Fidanza & Versiglioni, 1988) for most nutrients, and English tables or various Italian sources for the others (McCance & Widdowson, 1978). For all subjects a comulative average intake for each nutrient per day was computed by summing values for each food. Intakes of nutrients were then categorised into quintiles defined by daily consumption among controls.

The measure of association between cardia cancer risk and dietary and other factors was the odds ratio (OR) (Breslow & Day, 1980). For comparison, ORs were also calculated for other GCs. Both analyses utilised the same group of controls, including all non-resected subjects. To account for potential confounding by factors shown to be significantly related to total GC (Buiatti *et al.*, 1989*a*), multivariate stratified logistic regression analyses were conducted (SAS Institute, 1988). All the regression models included terms for age (actual age in years), sex, area (four study areas), place of residence (urban/rural), migration from the south (yes/no), socioeconomic status (low, medium and high, based on a combination of occupation and education), familial GC history (0, 1, 2+ first degree family members with GC), and Quetelet index (tertile categories of weight/height squared; cut-off values: 23.4 and 26.3) along with the dietary or other variables of interest. Total caloric intake (log-scale) was also included in each model when nutrient intakes were considered.

Correspondence: D. Palli, Epidemiology Unit, CSPO, Viale Volta 171, 50131 Florence, Italy.
Received 10 July 1991; and in revised form 15 October 1991.

### Results

Table I shows the distribution of the 923 cases of GC by site, age and sex. There were 68 (7.4%) cardia, 36 (3.9%) stump and 819 (88.7%) other site cancers. The sex ratios (M/F) were 2.8 for cardia, 11.0 for gastric stump, and 1.7 for other sites. Cardia tumours tended to arise at slightly younger age (median 63 *vs* 65 years). Table II shows the distribution of GC by sex and histologic type. In both sexes cardia tumours were less frequently classified as diffuse and more often as intestinal or unclassified compared to other sites. Table III shows the distribution of GC sites by area of residence. Because of a higher percentage of males and intestinal type tumours in high-risk areas, the proportion of cardia cancers was slightly higher in high-risk than in low-risk areas (7.7% *vs* 5.5%).

The ORs for cardia and other sites were estimated according to demographic and other variables. Because only 36 cases were classified as stump cancers, they were excluded from further analysis, together with all controls who reported previous gastric surgery. For each site, risks decreased with increasing social class, and were higher in residents of rural than urban areas. The risk of other GCs was significantly lower among persons migrating from the low-risk areas of southern Italy, but no such effect was seen for cardia cancer (OR = 1.3; 95% CI 0.5–3.4).

The risks for cardia and other GCs were elevated in association with a familial history of GC (Table IV). The risks were highest for cardia tumours, although not significantly different from other sites; four of the five cardia cases reporting two or more first degree relatives with GC were classified as intestinal according to Lauren, and one was unclassified. The risks for cardia and other GCs declined as the body mass index (weight/height-squared) increased.

Our earlier analyses (Buiatti *et al.*, 1989*a*) linked GC to certain diet-related variables, including ownership of a freezer (yes/no), age at which a refrigerator was obtained, habit of storing foods in the refrigerator (often/seldom), usual consumption of frozen foods (low, medium, high), and habit of adding salt (never/often) and taste for foods (low salt/normal/salty). Separate analyses of these variables revealed similar ORs for cardia *vs* other GCs, although a stronger increase in risk for cardia tumours was associated with preference for normal (OR = 3.3; 95% CI 1.1–9.3) or salty foods (OR = 3.2; 95% CI 1.1–9.9) *vs* low salt foods.

Table V shows ORs for the highest compared to the lowest tertile of intake of 17 food groups. Each food group was included in a separate model, adjusting for the matching variables and the main confounders. The ORs were similar for cardia and other GCs, showing increased risks associated with the consumption of traditional soups, meat and salted/dried fish, and decreased risks associated with raw vegetables, citrus and other fresh fruit.

Table VI presents the ORs associated with smoking and alcohol consumption. Cigarette smoking was evaluated using

**Table I** Distribution of gastric cancer cases by age, sex and site

| | | Cardia | | Stump | | All others | | |
|---|---|---|---|---|---|---|---|---|
| | Age | n | % | n | % | n | % | Total |
| *Males* | | | | | | | | |
| | <45 | 3 | (10.3) | 0 | (0) | 26 | (89.7) | 29 |
| | 45–54 | 5 | (5.6) | 3 | (3.3) | 82 | (91.1) | 90 |
| | 55–64 | 19 | (10.4) | 14 | (7.6) | 150 | (82.0) | 183 |
| | 65 + | 23 | (7.8) | 16 | (5.4) | 256 | (86.8) | 295 |
| | Total | 50 | (8.4) | 33 | (5.5) | 514 | (86.1) | 597 |
| *Females* | | | | | | | | |
| | <45 | 0 | (0) | 0 | (0) | 14 | (100) | 14 |
| | 45–54 | 4 | (9.3) | 0 | (0) | 39 | (90.7) | 43 |
| | 55–64 | 7 | (8.6) | 1 | (1.2) | 74 | (90.2) | 82 |
| | 65 + | 7 | (3.7) | 2 | (1.1) | 178 | (95.2) | 187 |
| | Total | 18 | (5.5) | 3 | (0.9) | 305 | (93.6) | 326 |

**Table II** Distribution of 923 GC cases according to sex, histologic type and site

| Males | Intestinal (%) | Diffuse (%) | Mixed (%) | Unclassified (%) | |
|---|---|---|---|---|---|
| Cardia | 33 (66) | 4 (8) | 5 (10) | 8 (16) | 50 (100) |
| Stump | 19 (58) | 7 (21) | 1 (3) | 6 (18) | 33 (100) |
| All others | 299 (58) | 105 (20) | 35 (7) | 75 (14) | 514 (100) |
| Total | 351 (59) | 116 (19) | 41 (7) | 89 (15) | 597 |
| *Females* | *Intestinal (%)* | *Diffuse (%)* | *Mixed (%)* | *Unclassified (%)* | |
| Cardia | 10 (56) | 2 (11) | 2 (11) | 4 (22) | 18 (100) |
| Stump | 3 (100) | – (0) | – (0) | – (0) | 3 (100) |
| All others | 146 (48) | 98 (32) | 20 (7) | 41 (13) | 305 (100) |
| Total | 159 (49) | 100 (30) | 22 (7) | 45 (14) | 326 (100) |
| Total | 510 (55) | 216 (23) | 63 (7) | 134 (15) | 923 (100) |

**Table III** Distribution of gastric cancer cases according to anatomic site, and controls by study area

| | Cardia n | Site Stump n | All others n | Total n | Controls n |
|---|---|---|---|---|---|
| Area 1 | 22 | 16 | 294 | 332 | 371 |
| (%) | (6.6) | (4.8) | (88.6) | (100) | |
| Area 2 | 39 | 17 | 407 | 463 | 543 |
| (%) | (8.4) | (3.7) | (87.9) | (100) | |
| Area 3 | 2 | 0 | 51 | 53 | 137 |
| (%) | (3.8) | (0) | (96.2) | (100) | |
| Area 4 | 5 | 3 | 67 | 75 | 108 |
| (%) | (6.7) | (4.0) | (89.3) | (100) | |
| Total | 68 | 36 | 819 | 923 | 1159 |
| (%) | (7.4) | (3.9) | (88.7) | (100) | |

Areas 1: Forli, Imola, Cremona (highest risk); 2: Florence, Siena; 3: Genoa; 4: Cagliari (lowest risk).

**Table IV** ORs and 95% CI for gastric cancer by site associated with familial history of GC and Quetelet index

| | Cardia | | All others | |
|---|---|---|---|---|
| | OR[a] | 95% CI | OR[a] | 95% CI |
| Familial history[b] | | | | |
| None | 1.0 | – | 1.0 | – |
| 1 | 1.2 | (0.5–2.5) | 1.8 | (1.4–2.3) |
| 2 + | 7.8 | (2.6–23.3) | 4.9 | (2.6–9.3) |
| Quetelet index[c] | | | | |
| Low | 1.0 | – | 1.0 | – |
| Medium | 0.9 | (0.5–2.1) | 0.9 | (0.8–1.2) |
| High | 0.5 | (0.3–0.99) | 0.7 | (0.6–0.9) |

[a]ORs adjusted for variables included in the basic model (see text); [b]Number of first degree relatives reported with gastric cancer; [c]Tertiles (kg $mt^{-2}$).

**Table V** ORs and 95% CI for gastric cancer by site associated with highest compared to lowest tertile of consumption of various food groups

| | Cardia | | All others | |
|---|---|---|---|---|
| | OR[a] | 95% CI | OR[a] | 95% CI |
| Bread and pasta | 0.8 | (0.4–1.5) | 1.0 | (0.8–1.3) |
| Traditional soups | 2.8 | (1.3–6.0) | 2.4 | (1.9–3.1) |
| Meat | 1.9 | (1.0–3.7) | 1.7 | (1.4–2.2) |
| Cold cuts | 1.2 | (0.6–2.4) | 1.3 | (1.0–1.7) |
| Salted/dried fish | 1.7 | (0.9–3.1) | 1.5 | (1.2–1.8) |
| Other fish | 0.7 | (0.4–1.2) | 1.0 | (0.8–1.3) |
| Milk/dairy products | 1.1 | (0.9–1.3) | 1.1 | (1.0–1.2) |
| Seasoned cheeses | 1.0 | (0.5–2.0) | 1.2 | (1.0–1.6) |
| Raw vegetables | 0.4 | (0.2–0.8) | 0.6 | (0.3–0.8) |
| Cooked vegetables | 1.5 | (0.8–2.8) | 1.1 | (0.9–1.4) |
| Beans | 1.0 | (0.5–1.8) | 0.8 | (0.6–1.0) |
| Spices | 0.8 | (0.4–1.6) | 0.7 | (0.5–0.8) |
| Onion/garlic | 0.7 | (0.3–1.4) | 0.8 | (0.6–1.0) |
| Citrus fruit | 0.3 | (0.2–0.6) | 0.6 | (0.4–0.7) |
| Other fresh fruit | 0.2 | (0.1–0.5) | 0.4 | (0.3–0.6) |
| Dried fruit | 0.8 | (0.4–1.6) | 1.0 | (0.8–1.2) |
| Desserts | 0.5 | (0.3–1.0) | 0.9 | (0.7–1.1) |

**Table VI** ORs and 95% CI for gastric cancer by site associated with consumption of wine, alcohol intake and smoking history

| | Cardia | | All others | |
|---|---|---|---|---|
| | OR[a] | 95% CI | OR[a] | 95% CI |
| Wine consumption[b] | | | | |
| Never | 1.0 | – | 1.0 | – |
| Less than twice/day | 0.8 | (0.3–2.3) | 0.8 | (0.6–1.2) |
| Twice/day or more | 1.3 | (0.6–2.6) | 1.2 | (0.9–1.5) |
| Alcohol intake | | | | |
| Low (lowest quintile) | 1.0 | – | 1.0 | – |
| High (highest quintile) | 1.4 | (0.7–3.7) | 1.0 | (0.7–1.2) |
| Smoking history | | | | |
| Never | 1.0 | – | 1.0 | – |
| Current | 1.1 | (0.6–2.3) | 0.9 | (0.7–1.1) |
| Ex-smoker | 1.1 | (0.5–2.2) | 1.1 | (0.8–1.4) |

[a]Adjusted for the variables in the basic model but not other food groups. [b]Times per day.

categories of never, current and ex-smokers, along with an index of pack-years of smoking among current and ex-smokers. Among current- or ex-smokers no increased risks of either cardia or other GCs were found. Results were similar when using other smoking history variables, such as pack-years. No significant increase in risk was shown with increasing consumption of wine. For cardia tumours, however, risk was highest (OR = 1.4; 95% CI 0.7–3.7) among those in the highest quintile of total alcohol intake.

Although they reported lower body mass indices in the period investigated by the questionnaire (2 years before the interview), the cases with other GCs tended to consume more calories than did controls, while no relation was seen for cardia tumours. In Table VII, ORs adjusted for caloric intake are shown for the highest compared to the lowest quintile of consumption of 12 nutrients. The ORs were generally similar for each site, with protective effects for ascorbic acid and alpha-tocopherol, and increased risks for the intake of protein, cholesterol, nitrites and starches. However, confidence intervals tended to be wider for cardia cancer.

Table VIII examines risks associated with animal *vs* vegetable fat, and animal *vs* vegetable protein. For both sites, an elevated risk was associated with high total protein intake (due entirely to animal protein), while a protective effect was associated with high vegetable fat intake.

Table IX shows a multivariate analysis in which the effects of dietary factors are adjusted for other factors. Included in the model were potential risk factors involved in nitrosation (protein and nitrites), and potential inhibitors in the form of anti-oxidants (ascorbic acid, beta-carotene and alpha-tocopherol). Vegetable fats were not included, because of their colinearity with alpha-tocopherol. In this multivariate model, decreased ORs were associated with ascorbic acid and alpha-tocopherol for both cardia and other GCs. Protein intake was a strong risk factor for both sites, while no association was shown with nitrite and beta-carotene.

The small number of cardia tumours precluded a meaningful analysis by histologic type, either for dietary or familial histories.

**Table VII** ORs and 95% CI for gastric cancer by site associated with highest compared to lowest quintile of intake of various nutrients

| | Cardia | | All others | |
|---|---|---|---|---|
| | OR[a] | 95% CI | OR[a] | 95% CI |
| Protein | 3.6 | (1.0–13.4) | 2.5 | (1.5–4.1) |
| Ascorbic acid | 0.4 | (0.2–0.8) | 0.5 | (0.3–0.6) |
| Alpha-tocopherol | 0.7 | (0.3–1.6) | 0.5 | (0.4–0.7) |
| Beta-carotene | 0.8 | (0.4–1.6) | 0.6 | (0.5–0.8) |
| Retinol | 1.9 | (0.9–4.1) | 1.1 | (0.8–1.4) |
| Nitrites | 1.4 | (0.5–4.1) | 2.0 | (1.3–2.9) |
| Nitrates | 1.1 | (0.6–2.3) | 0.7 | (0.6–1.0) |
| Fat | 1.2 | (0.4–3.0) | 0.5 | (0.3–0.8) |
| Cholesterol | 3.2 | (1.4–7.3) | 1.2 | (0.9–1.7) |
| Carbohydrates | 0.4 | (0.1–1.5) | 1.0 | (0.6–1.6) |
| Starches | 1.6 | (0.5–4.7) | 1.7 | (1.2–2.6) |
| Calcium | 0.9 | (0.4–2.1) | 1.0 | (0.7–1.3) |

[a]Adjusted for the variables in the basic model and kilocalories (log-scale), but not other nutrients.

**Table VIII** ORs and 95% CI for gastric cancer by site associated with highest compared to lowest quintile of intake of pairs of selected nutrients

| | Cardia | | All others | |
|---|---|---|---|---|
| | OR[a] | 95% CI | OR[a] | 95% CI |
| Animal protein[a] | 2.6 | (1.2–5.8) | 1.9 | (1.4–2.5) |
| Vegetable protein[a] | 0.5 | (0.2–1.2) | 1.0 | (0.8–1.4) |
| Animal fat[b] | 2.1 | (1.0–4.3) | 1.9 | (1.4–2.5) |
| Vegetable fat[b] | 0.5 | (0.3–1.1) | 0.6 | (0.4–0.8) |

[a]ORs derived from a logistic model including terms for the variables in the basic model plus kilocalories (log-scale), animal and vegetable proteins; [b]ORs derived from a logistic model including terms for the variables in the basic model plus kilocalories (log-scale), animal and vegetable fats.

**Table IX** ORs and 95% CI for gastric cancer by site associated with highest compared to lowest quintile of intake of five selected nutrients

| | Cardia | | All others | |
|---|---|---|---|---|
| | OR[a] | 95% CI | OR[a] | 95% CI |
| Protein | 5.1 | (1.2–21.4) | 3.1 | (1.8–5.3) |
| Nitrites | 0.9 | (0.3–2.7) | 1.2 | (0.8–1.9) |
| Ascorbic acid | 0.3 | (0.1–0.7) | 0.5 | (0.4–0.7) |
| Beta-carotene | 1.1 | (0.5–2.7) | 0.9 | (0.6–1.2) |
| Alpha-tocopherol | 0.8 | (0.3–2.1) | 0.7 | (0.5–0.97) |

[a]ORs derived from logistic models including terms for the variables in the basic model plus kilocalories (log-scale) and the five nutrients (log-scale).

### Discussion

In this population-based case-control study of GC in high-risk and low-risk areas of Italy, the number of subjects was sufficiently large to evaluate risk factors according to anatomic site. The cardia tumours, which represented less than 10% of the total, occurred proportionally more often in men than women, and were more frequently intestinal or unclassified and less often diffuse, when compared to distal sites in both sexes. Risk factors for cardia and other tumours were found to be very similar. No effect was found for smoking at either site and only a weak association with alcohol consumption was present for cardia tumours. Cardia tumours also showed a greater familial occurrence of GC than did other sites, but the difference was not statistically significant.

Our findings are interesting in view of the rising trend in the incidence rates for adenocarcinomas of the gastric cardia, gastro-esophageal junction and lower oesophagus that has been reported recently in the United States (Blot *et al.*, 1991; Yang & Davis, 1988) and northern Europe (Moeller & Jensen, 1987; Powell *et al.*, 1990), with cardia tumours representing up to 50% of the total GC incident cases among males. However, no appreciable trend over time has been observed for cardia tumours in the French-speaking Swiss canton of Vaud (Levi *et al.*, 1990). We do not have information on incidence trends for cardia cancer in Italy, but the relatively low percentage (7.4%) in our series suggests that, as of the mid-1980s, a large increase has not occurred. In our study the sex-ratio for cardia cancer was higher than for other sites, but not to the extent reported elsewhere (MacDonald & MacDonald, 1987; Yang & Davis, 1988). However, in the high-risk Japanese population, the sex-ratio (3.0) and prevalence (5.7%) for cardia tumours resemble what we have observed (Unakami *et al.*, 1989).

Few studies on gastric cancer have investigated risk factors according to anatomic site, including the cardia. We found no association with smoking nor with high wine consumption for cardia tumours, although a weak effect was suggested for total alcohol intake. In Japan, alcohol consumption was not associated with cardia or other GC tumours, but a smoking effect was suggested for cardia (Unakami et al., 1989). In China, alcohol consumption was too low to be evaluated, but smoking appeared to be weakly associated with cardia tumours (Li et al., 1989). In Los Angeles, a population-based study of young male GC patients (<55 years of age) noted an effect of alcohol consumption and smoking, most notably for cardia tumours (Wu-Williams et al., 1990). The consensus of evidence from all investigations, however, is that alcohol and tobacco consumption are at best only weakly related to cardia cancer, in contrast to the strong effects documented for squamous cell carcinoma of the oesophagus (Blot et al., 1991).

In our study risks associated with specific foods or food groups and nutrient intakes were remarkably similar when comparing cardia and distal tumours. These include the protective effects of raw vegetables, citrus and other fresh fruit and ascorbic acid, along with the risks imparted by the consumption of traditional soups and meat, as well as protein and a preference for salty foods. In Los Angeles, a significantly increased risk was associated with high consumption of beef for cardia but not other GCs (Wu-Williams et al., 1990). However, in China, where consumption of meat is low, no clear dietary factor was found for cardia tumours (Li et al., 1989).

In summary, this population-based case-control study of GC in Italy revealed no evidence that specific nutritional or other risk factors differentially affected the cardia vs distal sites. However, the proportion of cardia tumours is much smaller than in other western countries that have recently experienced remarkable increases in the incidence of adenocarcinomas of the cardia and lower oesophagus. Further research on environmental and host determinants of these emergent tumours is urgently needed.

This study was supported by the Consiglio Nazionale delle Ricerche – Applied project Oncologia, contracts 87.01506.44/87.01581.44/87.1344.44; the US National Cancer Institute, contract N01-CP-51019; the Istituto Oncologico Romagnolo, Forli, Italy; Regione Emilia-Romagna, Italy; Regione Toscana, Italy; Lega Italiana per la Lotta Contro i Tumori, Rome, Italy.

### References

BLOT, W.J., DEVESA, S.S., KNELLER, R.W. & FRAUMENI, J.F. Jr (1991). Rising incidence of adenocarcinoma of the esophagus and gastric cardia. *JAMA*, **265**, 1287.

BRESLOW, N.E. & DAY, N.E. (1980). *Statistical Methods in Cancer Research*. Vol. 1. The analysis of case-control studies. IARC Scientific Publication 32, IARC, Lyon.

BUIATTI, E., PALLI, D., DECARLI, A. & 11 others (1989*a*). A case-control study of gastric cancer and diet in Italy. *Int. J. Cancer*, **44**, 611.

BUIATTI, E., PALLI, D., AMADORI, D. & 9 others (1989*b*). Methodological issues in a multicentric study of gastric cancer and diet in Italy: study design, data sources and quality controls. *Tumori*, **75**, 410.

BUIATTI, E., PALLI, D., DECARLI, A. & 14 others (1990). A case-control study of gastric cancer and diet in Italy: II. Association with nutrients. *Int. J. Cancer*, **45**, 896.

BUIATTI, E., PALLI, D., BIANCHI, S. & 12 others (1991). A case-control study of gastric cancer and diet in Italy: III. Risk patterns by histologic type. *Int. J. Cancer*, **48**, 369.

FIDANZA, F. & VERSIGLIONI, N. (1988). Tabelle di composizione degli alimenti. In Fidanza, F. & Liguori, G. (eds), *Nutrizione Umana*, p. 677. Idelson, Naples.

HOWSON, C.P., HIYAMA, T. & WYNDER, E.L. (1986). The decline in gastric cancer: epidemiology of an unplanned triumph. *Epidemiol. Rev.*, **8**, 87.

LAUREN, P. (1965). The two histological main types of gastric carcinoma: diffuse and so-called intestinal-type carcinoma. *Acta Path. Microbiol. Scandinav.*, **64**, 31.

LEVI, F., LA VECCHIA, C. & TE, V.C. (1990). Descriptive epidemiology of adenocarcinomas of the cardia and distal stomach in the Swiss canton of Vaud. *Tumori*, **76**, 167.

LI, J., ERSHOW, A.G., CHEN, Z. & 5 others (1989). A case-control study of cancer of the esophagus and gastric cardia in Linxian. *Int. J. Cancer*, **43**, 755.

MACDONALD, W.C. & MACDONALD, J.B. (1987). Adenocarcinoma of the esophagus and/or gastric cardia. *Cancer*, **60**, 1094.

MCCANCE & WIDDOWSON (1978). *The Composition of Foods*, 4th revised edition, Paul, A.A. & Southgate, D.A.T. (eds), HMSO: London.

MOELLER, H. & JENSEN, O.M. (1987). Den tidsmÆssige udvikling i esophagus-, cardia- og ventrikelcancer i Danmark 1943–1982. *Ugeskr. LÆger.*, **149**, 1904.

PALLI, D., BIANCHI, S., CIPRIANI, F. & 11 others (1991). Reproducibility of histopathologic classification of gastric cancer. *Br. J. Cancer*, **63**, 765.

POWELL, J. & MCCONKEY, C.C. (1990). Increasing incidence of adenocarcinoma of the gastric cardia and adjacent sites. *Br. J. Cancer*, **62**, 440.

SAS INSTITUTE (1988). The CATMOD Procedure. In *SAS/STAT User's Guide*, release 6.03 Edition, pp. 189. SAS Institute: Cary, NC.

UNAKAMI, M., HARA, M., FUKUCHI, S. & AKIYAMA, H. (1989). Cancer of the gastric cardia and the habit of smoking. *Acta Pathol. Jpn.*, **39**, 420.

WU-WILLIAMS, A.H., YU, M.C. & MACK, T.M. (1990). Life-style, workplace, and stomach cancer by sub-site in young men of Los Angeles County. *Cancer Res.*, **50**, 2569.

YANG, P.C. & DAVIS, S. (1988). Epidemiological characteristics of adenocarcinoma of the gastric cardia and distal stomach in the United States, 1973–1982. *Int. J. Epidemiol.*, **17**, 293.